# Tissue polypeptide antigen & interleukin-6: Are their serum levels a predictor for response to chemotherapy in breast cancer?

**DOI:** 10.12669/pjms.305.5199

**Published:** 2014

**Authors:** Wahda Basheer Al-Youzbaki, Niaam Basheer Al-Youzbaki, Muwaffaq M Telfah

**Affiliations:** 1 Dr. Wahda Basheer Al-Youzbaki, MBChB; MSc; PhD Pharmacology, Assist. Prof., Head of Dept. of Pharmacology, College of Medicine, University of Mosul, Mosul, Iraq.; 2 Dr. Niaam Basheer Al-Youzbaki, MBChB; MSc; Microbiology, Assist. Lecturer, Dept. of Microbiology, College of Medicine, University of Mosul, Mosul, Iraq.; 3 Dr. Muwaffaq M Telfah, MBChB; MSc; MRCS/Eng, Lecturer, Dept. of Surgery, College of Medicine, University of Mosul, Mosul, Iraq.

**Keywords:** Breast cancer, Tumor marker, Interleukin, Tissue polypeptide antigen

## Abstract

***Objective:*** To compare the serum level of tissue polypeptide antigen (TPA) and interleukin-6 (IL-6) in women with breast cancer at various stages of the disease and to consider the usefulness of these tumor markers in evaluating the response to chemotherapy.

***Methods:*** This case control study included 60 women, from those who were attending the Oncology and Nuclear Hospital in Mosul / Iraq from the period of 1^st^ of March 2012 and 1^st^ of March 2013, complaining of breast cancer of stage 1-4 and receiving chemotherapy after they were operated on. Thirty women age ranged between 29-69 years, were receiving six cycles of chemotherapy after they were operated. This group was compared with the second group of 30 breast cancer women, age ranged between 28-61 years who came for the first time after they were operated on and before receiving chemotherapy. Another 30 apparently healthy, age matched women were included in this study as a healthy control group. The sera obtained from the precipitants used for the estimation of serum TPA and IL-6 level using special commercial kits.

***Results:*** The mean serum levels of both TPA and IL-6 were significantly higher in breast cancer patients than healthy control group. The mean serum levels of both TPA and IL-6 in the breast cancer patients who received 6 cycles of chemotherapy were significantly lower than their levels in the breast cancer patients who did not received chemotherapy yet. There was a significant difference among the 4 stages of breast cancer regarding TPA and IL-6 serum levels, the highest value was detected in those with stage IV and the lowest value was detected in those with stage I. The mean serum levels of both TPA and IL-6 were significantly higher in patients with ductal type than those with lobular type in both breast cancer groups. Both TPA and IL-6 are highly sensitive in detecting breast cancer and the combination of the two tumor markers will increase the specificity for detecting breast cancer up to 96.7%.

***Conclusion:*** Serum level of TPA and IL-6 discriminates between localized and metastatic breast cancer and their levels are good indicators of disease progression, TPA and IL-6 levels have a good predictive value for response to chemotherapy. The combination of the two tumor markers will increase the specificity for detecting breast cancer up to 96.7%.

## INTRODUCTION

Breast cancer is the most common type of cancer and the primary cause of cancer mortality in women.^1^ The main clinical application of tumor markers in breast cancer is in chemotherapy monitoring.^2^^,^^3^ In contrast to tissue markers, blood tumor markers reflect a dynamic situation and have the advantage that their measurements can be repeated easily as often as required.^4^ Nicolini *et al.,* (2006)^5^ emphasized that the inclusion of serum tumor markers is an important factor in the postoperative monitoring of breast cancer patients. The European Group on Tumor Markers (EGTM) panel^6^ considers a decrease of more than 50% in the serum levels of tumor marker indicates response to chemotherapy. Certain treatment regimens may cause transient increases in serum marker levels, a phenomenon known as "spiking".^7^

Tissue polypeptide antigen (TPA) is a complex of polypeptide filaments of the cytokeratins 8, 18 and 19 and is produced during late S and G2 phases of the cell cycle. TPA represents the most abundant cytokeratins pattern in malignant epithelial differentiation.^8^ The moderate elevation in TPA occurs in some benign events such as liver failure, renal failure, diabetes mellitus and pregnancy.^9^ The marked elevation of serum TPA is reported in variety of cancers such as breast, lung, gastrointestinal, urological, gynecological cancer**,** therefore serum level of TPA is valuable as a prognostic marker and for monitoring treatment of patients with different carcinomas.^10^

Interleukin 6 (IL‐6) is a proinflammatory cytokine, which is produced by a number of immune system cells; fibroblasts, macrophages, T and B Lymphocytes, endothelial cells, keratinocytes and tumor cells.^11^ It may play a role in the proliferation and metastasis of cancer by up regulating antiapoptotic and agiogenic proteins in tumor cells.^12^ The study by Salgado *et al.,*^13^ reported that there is a prognostic significance for serum IL-6 measured at the time of diagnosis of metastasis. High serum levels of IL-6 correlate with poorer outcomes in breast cancer patients.^14^ The aim of this study was to compare the serum level of TPA, and IL-6 in women with breast cancer at various stages of the disease and to consider the usefulness of these tumor markers in evaluating the response to chemotherapy for these patients.

## METHODS

The approval of the study protocol by an ethic committee has been obtained from the local health committee of Ministry of Health and College of Medicine – University of Mosul – Iraq. This was a case-control study which included 60 women from those who were attending the Oncology and Nuclear medicine Hospital in Mosul / Iraq from the period of 1^st^ of March 2012 and 1^st^ of March 2013, complaining of breast cancer of stage 1-4, their diagnosis depending on clinical and histopathological findings depending on TNM staging system.^15^ Thirty of these women, age ranged between 29-69 years, were receiving six cycles of chemotherapy after they were operated on. This group was compared with the second group of the other 30 breast cancer women, age ranged between 28-61 years who came for the first time, 3 weeks after they were operated on and before receiving chemotherapy. Another 30 apparently healthy, age matched women were included in this study as a healthy control group.

Blood samples from first group were collected after receiving the 6^th^ cycle of chemotherapy and from the second group 3 weeks postoperatively and before receiving the first cycle of chemotherapy. The separated sera were used for measurement of TPA and IL-6 concentration using two enzyme linked immunosorbent assay (ELISA) kits, one for TPA detection was supplied by DRG instruments GmbH, Germany The other ELISA kit for IL-6 detection supplied by RayBiotech, Inc.

Standard statistical methods were used to determine the mean, standard deviation (SD) and the range. Independent two samples student- t test and ANOVA test with post hoc Waller- Duncan test were used. All values quoted as the mean ± SD and a P-value of < 0.05 was considered to be statistically significant.

## RESULTS

Levels of TPA and IL-6 were assessed in the sera of the blood of 30 patients with breast cancer, with mean age ±SD of (46.91±8.65 years), after receiving six cycles of chemotherapy and compared to their values in another 30 breast cancer patients with mean age ±SD of (45.43±7.99 years), 3 weeks postoperative and just before receiving the first cycle of chemotherapy. The serum level of TPA and IL-6 in both breast cancer patients groups were compared to their values in another 30 healthy women age ranged between 28-65 years and considered as a healthy control group. Table-I demonstrates the general characteristics of the two groups of the breast cancer patients.

**Table-I:** General characteristics of the two groups of breast cancer patients

*Variables*	*Control group of breast cancer patients (before chemotherapy)* *n= 30*	*Breast cancer patients after 6 cycles of chemotherapy* *n= 30*
**Age in years (mean ± SD)**	**46.91 ± 8.65 years**	**45.43 ± 7.99 years**
***Menopausal status:***		
**Premenopausal**	**18 (60%)**	**20 (66.66%)**
**Postmenopausal**	**12 (40%)**	**10 (33.33%)**
***Clinical stage:***		
**Stage I (T1-2N0M0)**	**3 (10%)**	**2 (6.66%)**
**Stage II (T1-2 N1M0)**	**7 (23.33%)**	**5 (16.66%)**
**Stage III(T1-3 N0 2M0**	**15 (50%)**	**18 (60%)**
**Stage IV(T1-3 N0-2M)**	**5 (16.66%)**	**5 (16.66%)**

T: Tumour size; T1 .2cm; T2 .2.4cm; T3 .4cm.

N: Nodal metastasis; N0 = No regional lymph node metastasis.

N1: Metastasis in a single ipsilateral node of <3cm diameter.

N2: Metastasis in a single ipsilateral node of .3cm diameter.

M: Distant metastasis; M0 = No distant metastasis.

Table-II demonstrates that the mean serum levels of both TPA and IL-6 were significantly higher in breast cancer patients than healthy control group.

**Table-II:** Comparison between mean serum TPA and IL-6 levels of breast cancer patients & healthy controls group

***Parameters***	***Breast cancer patients n=60***	***Healthy control group n=30***	***P-value***
TPA ng/ml	18.94 ±13.51	3.11 ± 3.94	<0.0001
IL-6 pg/ml	57.38 ± 48.62	6.91 ± 4.31	<0.0001

Table-III shows that the mean serum levels of both TPA and IL-6 in the first group of breast cancer patients who received 6 cycles of chemotherapy were significantly lower than their levels in the control group of breast cancer patients who did not received chemotherapy yet.

**Table-III:** Comparison between serum TPA and IL- 6 levels in the two groups of the breast cancer patients

***Parameters***	***Mean ± SD***		***p-value***
	***Breast cancer patients not receiving chemotherapy yet (n=30)***	***Breast cancer Patients after receiving 6 cycles of chemotherapy (n=30)***	
TPA (ng/ml)	28.58 ± 17.24	14.27 ± 7.98	<0.0001
IL-6 (pg/ml)	91.86 ± 59.96	39.54 ± 28.80	<0.0001

Table-IV illustrates that the highest value of TPA and IL-6 was detected in breast cancer patients with stage IV and the lowest value was detected in those with stage I. There was a significant difference among the 4 stages of breast cancer regarding TPA and IL-6 serum levels.

**Table-IV:** Serum TPA and I-L6 levels at different clinical stages of the two groups of the breast cancer patients

***Clinical stage of breast cancer***	***Breast cancer patients*** ***Total number =60***	***TPA ng/ml*** ***Mean±SD***	***IL-6 pg/ml*** ***Mean±SD***
Stage I	5 (8.3%)	12.70±1.82	7.40±7.99
Stage II	12 (20%)	13.68±8.15	31.25±14.51
Stage III	33 (55%)	16.05±7.81	44.73±29.45
Stage IV	10 (16.7%)	35.22±17.66	127.52±52.23
p-value		< 0.0001	< 0.0001

Table-V shows that the mean serum levels of both TPA and IL-6 were significantly higher in patients with ductal type of breast cancer than those with lobular type in both breast cancer groups.

**Table-V:** Relationship between mean serum TPA and IL-6 levels & histopathological type of breast cancer

***Parameters***	***Mean ± SD***		***p-value***
	***Ductal breast cancer*** ***(n=47) (78.33 %)***	***Lobuler breast cancer*** ***(n=13) (21.66 %)***	
TPA (ng/ml)	20.61±14.47	13.160±7.15	0.02
IL-6 (pg/ml)	66.00±51.25	28.07±19.85	0.002

Table-VI illustrates that the positive predictive value (PPV) of IL-6 is more than TPA and the combination of the two is more. Also both TPA and IL-6 serum level are highly sensitive in detecting breast cancer and the combination of the two tumor markers will increase the specificity for detecting breast cancer up to 96.7%.

**Table-VI:** The diagnostic validity parameters of TPA and IL-6 in breast cancer

***Parameters***	***Sensitivity***	***Specificity***	***PPV***	***NPV***	***Accuracy***
TPA ng/ml	96.7%	53.3%	86.1%	84.2%	85.8%
IL-6 pg/ml	87.8%	83.3%	94.0%	69.4%	86.6%
TPA + IL-6	85.6%	96.7%	98.7%	69.0%	88.3%

## DISCUSSION

In this study, a significant high serum level of TPA was detected in breast cancer patients compared with healthy control group but significant lower TPA serum level in breast cancer patients who received 6 cycles of chemotherapy than those who did not received chemotherapy yet (Table II and III), which mean that serum TPA level have a good predictive value for response to chemotherapy and normally decrease in response to successful treatment. This results is in accordance with a study done by Sjostrom and colleagues (2001).^16^

The evaluation of serum samples obtained from 60 women with breast cancer revealed higher TPA serum levels in cases with more advanced disease (stage III and IV) than those with localized breast cancer (Table-IV) which indicates that high levels of TPA appear to be related to the tumor burden. This result is similar to a study done by Sliwowska *et al.,*^17^ which found that TPA level correlates well with clinical stages of breast cancer.

Tissue polypeptide antigen (TPA) is one of the proposed tumor markers for monitoring therapy in patients with breast cancer beside carcinoma antigen 15.3 (CA15.3), carcinogenic embryonic antigen (CEA), tissue polypeptide specific antigen (TPS) and circulating tumor cells (CTC).^18^ Although the main use of cytokeratins like TPA is to monitor treatment and evaluate response to therapy, early prognostic information particularly on tumor progression and metastasis formation is also provided for several types of cancers and they offer a simple, noninvasive, cheap, and reliable tool for more efficient management.^19^

This study showed that serum IL-6 level was significantly lower in patients who had received 6 cycles of chemotherapy than those who did not received chemotherapeutic agents (Table-III), which indicates that IL-6 level is a reliable predictive marker for response to chemotherapy in breast cancer patients. This result goes in agreement with another research^20^ which reported that compared to pre-treatment, radiotherapy and/or chemotherapy for breast cancer led to a significant reduction in circulating IL-6 level at 3 months and at 12 months following treatment. Another study done by Mills and his Colleagues (2008) ^21^ demonstrated that 3 cycles of chemotherapy for breast cancer lead to elevations in inflammatory markers including IL-6, associated with endothelial and platelets activation. This may be explained by (spiking phenomenon), that is some chemotherapy regimens, the first 2 or 3 cycles may lead to transient increases in serum markers levels.^7^

In this study, a significant high serum level of IL-6 was detected in breast cancer patients compared with healthy control group, with direct association to clinical stages Table-II and IV. Similar results were found by other researchers.^22^^,^^23^ In addition, another study was done by Beny and his Colleagues (2002),^24^ showed a 10 times increase in IL‐6 level in metastasis breast cancer patients compared with local site disease. This is probably related to the fact that IL-6 can help tumor to grow through the inhibition of cancer cells apoptosis and the induction of tumor angiogenesis.^12^ Hence there is an interest in developing anti-IL-6 agents as therapy against tumors.^25^^,^^26^

Regarding histological pattern of breast cancer, invasive ductal carcinoma was the most common type which was seen representing (78.33%) (Table-V). The remaining patients had lobular carcinoma which represented (21.66 %) of the 60 breast cancer patients included in this study. This result was in accordance to other reports.^27^^,^^28^ The patients with invasive ductal carcinoma had higher serum levels of TPA and IL-6 than those with lobular carcinoma. This suggests that ductal breast cancer had a worse prognosis than lobular carcinoma. This goes in agreement with other researches which revealed that despite developments in surgical methods, cytotoxic chemotherapy, and targeting agents against estrogen receptor and HER2, a subset of patients with advanced-stage invasive ductal carcinoma display poor prognosis and early metastasis after single or combination treatment. An estimated 11% of women with invasive ductal carcinoma will experience recurrence within five years after surgery.^29^^,^^30^

In the diagnosis of breast cancer, the TPA test had a greater diagnostic sensitivity (96.7%) in detecting breast cancer compared to that of IL-6 test (87.8%) (Table-VI). This result is in agreement with another study^17^ which revealed that the TPA had the greatest diagnostic value in detecting breast cancer compared to CA15.3 and TPS. However, IL-6 serum level had a higher diagnostic specificity (83.3%) than that of the TPA level (53.3%). Furthermore, the combination of both serum TPA and IL-6 levels have found to increase the specificity in detecting breast cancer up to (96.7%) and up to authors knowledge this is the first study estimated the specificity and sensitivity of the combination of these tumor markers in the diagnosis and follow up of patients with breast cancer on chemotherapy. Thus, there may be a place to add serum TPA and IL-6 to the preoperative diagnostic tools, especially in cases of difficult decision between benign changes of the breast and disseminated carcinoma in situ (DCIS) on mammography.

## Conclusions

1. TPA and IL-6 levels discriminate between localized and metastatic breast cancer and their levels are good indicators of disease progression,
2. TPA and IL-6 levels have a good predictive value for response to chemotherapy and usually decrease in response to successful treatment. If TPA and IL-6 levels remain unaffected or increase, a change of treatment should be considered.
3. Both TPA and IL-6 are highly sensitive in detecting breast cancer and the combination of the two tumor markers will increase the specificity for detecting breast cancer up to 96.7%.

## Authors Contribution:

All the authors have equally contributed to the conduction of this study and writing the manuscript. Authors of this study had full excess to all the data and take complete responsibility for the integrity of the data and the accuracy of the data analysis.

## References

[1] Jemal A, Bray F, Center MM, Ferlay J, Ward E, Forman D (2011). Global cancer statistics. CA Cancer J Clin.

[2] Rao VSR, Dyer CE, Jameel JKA, Drew PJ, Greenman J (2006). Potential prognostic and therapeutic roles for cytokines in breast cancer (Review). Oncology Reports.

[3] Molina R, Auge JM, Avello N, Filella X (2006). Serum biomarkers in breast cancer. CLI.

[4] Nicolini A, Giardino R, Carpi A, Ferrari P, Anselmi L, Colosimo S (2006). Metastatic breast cancer: an updating. Biomed Pharmacother.

[5] Nicolini A, TartarelliG, Carpi A, Metelli M R, Ferrari P, Anselmi L (2006). Intensive post-operative follow-up of breast cancer patients with tumour markers: CEA, TPA or CA15.3 vs MCA and MCA-CA15.3 vs CEA-TPA-CA15.3 panel in the early detection of distant metastases. BMC Cancer.

[6] Molina R, Barak V, van Dalen A, Duffy MJ, Einarsson R, Gion M (Tumour Biol. 2005). Tumour markers in breast cancer. European Group on Tumour Markers Recommendations.

[7] Yasasever V, Dincer M, Camlica H, Karaloglu D, Dalay N (1997). Utility of CA 15-3 and CEA in monitoring breast cancer patientswith bone metastases: special emphasis on "spiking" phenomena. Clin Biochem.

[8] Ahn KS, Moon H, Ko E, Kim HS, Shin H, Kim J (2013). Preoperative serum tissue polypeptide-specific antigen is a valuable prognostic marker in breast cancer. IJC.

[9] Tramonti G, Pagano M, Galeazzi D (2000). Renal function and serum concentration of five tumor markers (TATI, SCC, CYERA21, TPA, TPS) in patients without evidence of neoplasia. Cancer Detection Prevention.

[10] Malati T (2007). Tumour markers: An overview. Indian J Biochem.

[11] Guo Y, Xu F, Lu T, Duan Z, Zhang Z (2012). Interleukin-6 signaling pathway in targeted therapy for cancer. Cancer Treat Rev.

[12] Backhelot T, Coquard IR, Menetrier- Caux C, Rastkha M, Duc A (2003). Prognostic value of serum level of interleukin 6 and of serum and plasma levels of vascular endothelial growth factor in hormone refractory metastatic breast cancer patients. Brit J Cancer.

[13] Salgado R, Junius S, Benoy I, Dam P V, Vermeulen P, Marck EV (2002). Circulating interleukin-6 predicts survival in patients with metastatic breast cancer. Int J Cancer.

[14] Barton BE (2005). Interleukin-6 and new strategies for the treatment of cancer, hyperproliferative diseases and paraneoplastic syndromes. Expert Opin Ther Targets.

[15] (2010 May 5). What is Cancer Staging?. http://www.cancerstaging.org/mission/whatis.html.

[16] Sjostrom J, Alfathan H, Joensuu H, Stenman UH, Lundin J, Blomquist C (2001). Serum tumor markers CA15-3, TPA, TPS, hCG beta and TATI in the monitoring of chemotherapy response in metastatic breast cancer. Scandin J Clin Lab Invest.

[17] Śliwowska I, Kopczyński Z, Grodecka-Gazdecka S (2006). Diagnostic value of measuring serum CA 15-3, TPA, and TPS in women with breast cancer. Postepy Hig Med Dosw.

[18] Sturgeon CM, Duffy MJ, Stenman UK, Lilja H, Brünner N, Chan DW (2008). National Academy of Clinical Biochemistry Laboratory Medicine practice guidelines for use of tumor markers in testicular, prostate, colorectal, breast and ovarian cancers. Clin Chem.

[19] Barak V, Gioke H, Panaretakis KW, Eianarsson R (2004). Clinical utility of cytokeratins as tumor markers. Clin Biochem.

[20] Caine GJ, Stonelake PS, Lip GY, Blann AD (2007). Changes in plasma vascular endothelial growth factor, angiopoietins, and their receptors following surgery for breast cancer. Cancer Lett.

[21] Mills PJ, Ancoli-Israel S, Parker B, Natarajan L, Hong S, Jain S (2008). , Predictors of inflammation in response to anthracycline- based chemotherapy for breast cancer. Brain Behay Immun.

[22] Suhail RAL (2008). Serum level of interleukin 6 and tumor necrosis factor in Iraqi breast cancer patients. MMJ.

[23] Zhang GT, Adahi I (2000). Interleukin 6 level correlate to tumor progression and prognosis in metastatic breast carcinoma. Anticancer Res.

[24] Beny I, Salgado R, Colparet C (2002). Serum VEGA and VEGF platelet load in breast Cancer patients. Clin Breast Cancer.

[25] Cho YA, Sung MK, JY Yeon JY, Ro J, Kim J (2013). Prognostic Role of Interleukin-6, Interleukin-8, and Leptin Levels According to Breast Cancer Subtype. Cancer Res Treat.

[26] Smolen JS, Maini RN (2006). Interleukin-6: a new therapeutic target. Arthritis Res Ther.

[27] Howlader NNA, Krapcho M, Neyman N, Aminou R, Waldron W, Altekruse SF (2011). SEER Cancer Statistics Review, 1975-2008, National Cancer Institute. http://seer.cancer.gov/csr/1975_2008/.

[28] Yasemi M, Ahmadi MH, Khajavikhan J, Peyman H, Asadollahi KH, Yasemi MR (2013). An 8 years retrospective study of breast cancer incidence in IIam, Province, Western Iran. J Clin Diagn Res.

[29] Voduc KD, Cheang MC, Tyldesley S, Gelmon K, Nielsen TO, Kennecke H (2010). Breast cancer subtypes and the risk of local and regional relapse. J Clin Oncol.

[30] Ozbay T, Nahta R (2011). Delphinidin inhibits HER2 and Erk1/2 signaling and suppresses growth of HER2-overexpressing and triple negative breast cancer cell lines. Breast Cancer.

